# The "Hot Potato" of Mental Health App Regulation: A Critical Case Study of the Australian Policy Arena

**DOI:** 10.15171/ijhpm.2018.117

**Published:** 2018-12-16

**Authors:** Lisa Parker, Lisa Bero, Donna Gillies, Melissa Raven, Quinn Grundy

**Affiliations:** ^1^Charles Perkins Centre, Faculty of Medicine and Health School of Pharmacy, The University of Sydney, NSW, Sydney, Australia.; ^2^NDIS Quality and Safeguards Commission, Penrith, NSW, Australia.; ^3^Critical and Ethical Mental Health Research Group, Robinson Research Institute, The University of Adelaide, Adelaide, SA, Australia.; ^4^Discipline of Public Health, Flinders University, Adelaide, SA, Australia.; ^5^Faculty of Nursing, University of Toronto, Toronto, ON, Canada.

**Keywords:** Mobile Applications, mhealth, Regulation, Policy Analysis, Mental Health, Australia

## Abstract

**Background:** Health apps are a booming, yet under-regulated market, with potential consumer harms in privacy and health safety. Regulation of the health app market tends to be siloed, with no single sector holding comprehensive oversight. We sought to explore this phenomenon by critically analysing how the problem of health app regulation is being presented and addressed in the policy arena.

**Methods:** We conducted a critical, qualitative case study of regulation of the Australian mental health app market. We purposively sampled influential policies from government, industry and non-profit organisations that provided oversight of app development, distribution or selection for use. We used Bacchi’s critical, theoretical approach to policy analysis, analysing policy solutions in relation to the ways the underlying problem was presented and discussed. We analysed the ways that policies characterised key stakeholder groups and the rationale policy authors provided for various mechanisms of health app oversight.

**Results:** We identified and analysed 29 policies from Australia and beyond, spanning 5 sectors: medical device, privacy, advertising, finance, and digital content. Policy authors predominantly framed the problem as potential loss of commercial reputations and profits, rather than consumer protection. Policy solutions assigned main responsibility for app oversight to the public, with a heavy onus on consumers to select safe and high-quality apps. Commercial actors, including powerful app distributors and commercial third parties were rarely subjects of policy initiatives, despite having considerable power to affect app user outcomes.

**Conclusion:** A stronger regulatory focus on app distributors and commercial partners may improve consumer privacy and safety. Policy-makers in different sectors should work together to develop an overarching regulatory framework for health apps, with a focus on consumer protection.

## Introduction

In 2016, the US Food and Drug Administration (FDA) stated it did not intend to regulate mobile applications (apps) that focused only general health and wellness.^1^ The news was welcomed by the digital health industry, which promptly expressed its approval at being “free[d]…from rules that could potentially slow progress in the field,”^2^ explaining that “regulations…can be time-consuming and expensive.”^3^ Dr. James Madara, CEO of the American Medical Association, was more cautious, suggesting that oversight was needed to reduce patient harm from the “scam[s]” and “digital snake oil” that currently exist within the “tsunami” of health apps.^4,5^ He agreed, however, that avoiding “heavy-handed” interference from government regulatory agencies in digital health was a good thing.^5^ A recent viewpoint by FDA leadership published in *the Journal of the American Medical Association* confirms this stance for the majority of health-related apps and signals their intention to harmonize regulations internationally.^6^

This recent policy development takes place in a time of rapid growth in the health app industry.^7^ There is enthusiasm among governments and clinicians about benefits, including improved self-care, prevention, enhanced accessibility, and cost-savings.^7-10^ However, there is also concern about the ability of health apps to collect, aggregate and share individuals’ data.^11-13^ Apps routinely, and legally, share de-identified users’ data with an ‘ecosystem’ of companies that aggregate, analyse, and commercialise this data into algorithms and insights.^14,15^ However, studies have shown that data is easily re-identified and used for highly targeted advertising, causing psychological harm^15^ or used to make decisions about individuals’ employability, insurability and financial health, whether or not the underlying data is accurate or complete.^14^ Other concerns about health apps pertain to the lack of clear evidence of health benefits.^16,17^ The vast majority of mental health apps, for example, have not been formally tested,^8,12,17^ and for the few that have, scientific trials have been small, with short follow-up.^18^ This may mean health app users experience financial and other costs without any offset of benefit.^8,12,17,18^ Apps may also cause direct harm to health.^13,19^ For example, apps that encourage self-monitoring and/or sharing may lead to anxiety in users who cannot keep up with recommended activities or find their results compare unfavourably with others.^20^

The explosion in the health app market has been accompanied by growing attention from policy-makers. Providing oversight is a demanding prospect for regulatory agencies given the emergent and rapidly changing nature of the industry, and the sheer volume of apps commercially available. Policy researchers have critiqued the current “patchwork”^5^ of regulatory oversight^21^ from medical device and privacy regulators and others. Many policies concentrate on what regulators do not intend to regulate.

Despite the level of interest in health apps and both realised and potential consumer harms, this arena remains under-regulated.^13,22,23^ We sought to understand this phenomenon by analysing how current policy solutions represented the problem of health app oversight. Further, we analyse the way policy authors characterise stakeholders within the health app policy arena, qualifying their degree of influence and responsibility.

## Methods

This study was a critical, qualitative case study of the policy arena around the mental health app market in Australia, designed in partnership with a consumer advocacy organisation, which served to identify priority consumer concerns. We defined policy as a: “set of goals, objectives and means that create a framework for activity.”^24^ We expected policies would include legislation, industry self-regulation, and post-market certification or evaluation programs.^25,26^ For the purposes of this project, we term the entities publishing policy as ‘policy authors.’

We selected the oversight of mental health apps as a policy case study because mental health apps are particularly illustrative of regulatory issues pertaining to all health apps. We use the term ‘health app’ to mean software focused on health and/or wellness that is designed to be downloaded onto a smartphone, tablet computer, or other mobile platform and run with or without internet availability.^27^ Mental health apps address one or more of a wide range of concerns ranging from mental illness (such as major depression or anxiety) to mental wellness (such as mindfulness).^28^ Mental health apps are prominent within the health app market,^29^ with tens of thousands of different products available.^30^ They have also garnered significant attention amongst academics and clinicians.^28,31,32^ The World Health Organization (WHO) and governments around the world are promoting digital technologies, including apps, as an accessible and cost-effective means of delivering mental healthcare.^9,33-35^ Mental health apps are subject to the same regulatory oversight as apps that focus on somatic health issues, however, while all health data is sensitive, mental health data is particularly so, highlighting the importance of mental health app user privacy.^8^ In addition, the wide ‘grey’ zone between mental health and illness highlights the difficulty of assessing the applicability of medical device regulation to many health apps.^19,31^

### Theoretical Framework

This analysis was informed by Bacchi’s ‘What’s the Problem Represented to be?’^36^ approach. In conventional understandings of policy, policymakers are seen to react to social problems that exist beyond the policy process. However, Bacchi’s approach traces policy solutions back to the kinds of problems they purport to address. The problem is rarely stated explicitly in a policy document, but is implied by the proposed policy ‘solutions.’ According to Bacchi, the way policies represent problems plays an active part in shaping societal understanding about the nature of problems. For example, proposing additional education for health app developers about the law helps shape a societal understanding that the problem of oversight is one of *knowledge deficit*, whereas requiring submission of safety data to a medical device regulator identifies the problem as a *risk of harm to health.* We also used Bacchi’s concept of subjectification, which proposes that policies influence the ways stakeholders are seen by themselves and others and presumes that the effects of particular problem representations may create hardships for some social groups more than others. For example, a policy solution may attribute responsibility to a particular social group, with negative effects for members of that group, while assigning benefits to a social group with a more favourable social construction.

### Sampling

Consistent with qualitative methodology, we purposively sampled for influential policies during September-October 2016 that addressed mental health app development, distribution, or selection for use, meaning that the sampling strategy was defined by a set of inclusion criteria specific to the context of the case study.^37^ We began sampling from the perspective of the Australian app market, which is one of the most concentrated smartphone markets in the world^38^ and a jurisdiction that is currently promoting the use of digital mental health services.^39^ However, the app market is global, as is the policy arena around health app oversight; thus, we also sampled policies from international jurisdictions that were referenced or relevant in the Australian context.

One investigator used the following sampling strategies to identify candidate policies for inclusion:

- Screening the websites of Australian government departments, industry trade associations, and mental health consumer organisations
- Medline and Google searches for policy literature using medical subject headings (MeSH) and keywords identified from relevant literature^17,20,27,40^: smartphone/cell phone/mobile applications/mobile phone/iPhone/mobile app/mobile health/mhealth AND mental disorders/mental health/psychotic disorders/bipolar disorders/schizophrenia/psychological stress/depression/depressive disorder/mood disorder/anxiety/anxiety disorder/mental illness/meditation/mindfulness/psychiatry AND health policy/policy/policy-making/public policy/framework/regulation/politics
- Hand searching through references and links in policies identified via steps 1 and 2;

We validated our search strategy by sharing a list of candidate policies with our research partners, including representatives of a peak telecommunications consumer advocacy group and digital health policy experts.

Two investigators independently screened the list of candidate policies for the following inclusion criteria:

- Pertain to apps on a mobile platform
- Apply to apps that provide information, diagnosis, monitoring, treatment, or support related to mental health
- Be published by an active, prominent entity defined as: a government; major university, hospital or mental healthcare institution; multinational corporation; or a national organisation, including peak bodies, and not-for-profit organisations
- Has an enforcement or inducement mechanism (such as a fine, exclusion from an industry body, or the ‘carrot’ of a quality certificate from a prominent entity)
- Clear evidence of implementation (eg, curated app libraries, promulgated legislation, active guidance)
- Publication in the last 10 years
- Australian focus or a clear influence on the Australian context defined as publication by an Australian policy author, jurisdiction over the Australian app market, or reference by an Australian policy document.

We excluded policies that were not freely available in English. Discrepancies related to inclusion were resolved through discussion until consensus was reached. The resulting list of policies was again circulated to a peak telecommunications consumer body and digital health policy experts to validate that all relevant policies and policy authors had been included, who confirmed there were no major omissions.

### Data Extraction and Analysis

We used the policies with associated websites and documents to source and record ancillary contextual information. This included: location and funding sources for the policy author (eg, government, non-profit, commercial); main role of the policy author; previous and related policies from the same author; policy author associates, informants, and target audience. We constructed an open-ended coding instrument to capture salient aspects within each policy, based on Bacchi’s policy theory.^36^ This included the target population and their subjectification, the ‘solution’ and implicitly defined ‘problem’ provided within each policy, tacit suppositions and judgements about the topic of mental health app regulation, and likely effects of the policy.

Each policy was read in detail and the first author wrote a preliminary memo that included a summary of the policy, contextual details, and definitions of unfamiliar terms. Researchers read the preliminary memo before coding the policy using the instrument described above and were instructed to iteratively incorporate contextual and related information where relevant. To enhance rigor, all policies and memos were reviewed by the senior author and a representative selection of policies were double coded. Any discrepancies were discussed, and resolved, erring on the side of comprehensive data interpretation. We presented preliminary interpretations to colleagues and policy partners, including an organisation representing consumers in the telecommunications industry and digital health policy experts and used these discussions to refine our findings.

## Results

We identified 29 policies, including guidance from government regulators, industry codes of conduct, and post-market app evaluation programs, mostly from non-Australian locations (see references S1-S29 in Supplementary file 1). All but one pertained to health apps in general. Ten policies (34%) were authored in Australia or New Zealand; 11 in the United States (38%); 5 in Europe (17%) and 3 (10%) by international bodies. These policies corresponded to 5 principal sectors: medical device, privacy, advertising, finance, and digital media content.

### A Framework for Policy Action

Based on our analysis, we devised a framework that describes the location of policy oversight in the health app field. According to our framework, any given policy shapes the app market at one or more different points along the trajectory, or ‘stream,’ of health app development and consumption, from inspiration, through development and distribution, to selection for use by consumers. We classified policies as ‘upstream’ (eg, government guideline), ‘midstream’ (eg, app store guidance for developers), or ‘downstream’ (eg, quality certification or collated library of preferred apps) according to their location along this trajectory (Table).

**Table:** Framework for Health App Policy

**Type of Policy ‘Barrier’**	**Mechanism and Location of Oversight**
Upstream	
eg, government guideline	• Defines and oversees the pool of legally compliant apps • Enacted by legislators and implemented by regulators • Includes legislation and regulatory guidance, which may not be legally binding but carry the influence of the regulator
Midstream	
eg, app store guidance for developers	• Defines the pool of apps that are commercially available to consumers • Due to the smartphone market dominance of Apple and Google, in practice this includes the iTunes and Google Play app store guidelines • Although compliance is voluntary, developers must submit their apps for review before they can be commercially distributed through these stores
Downstream	
eg, certification program for ‘high-quality’ apps according to pre-defined criteria	• Seeks to identify high-quality apps on the market as a signal to consumers • Achieves this through curation, endorsement, certification programs, or adherence to best practices • Compliance is voluntary and there are no formal consequences for non-compliance

**Figure 1:**
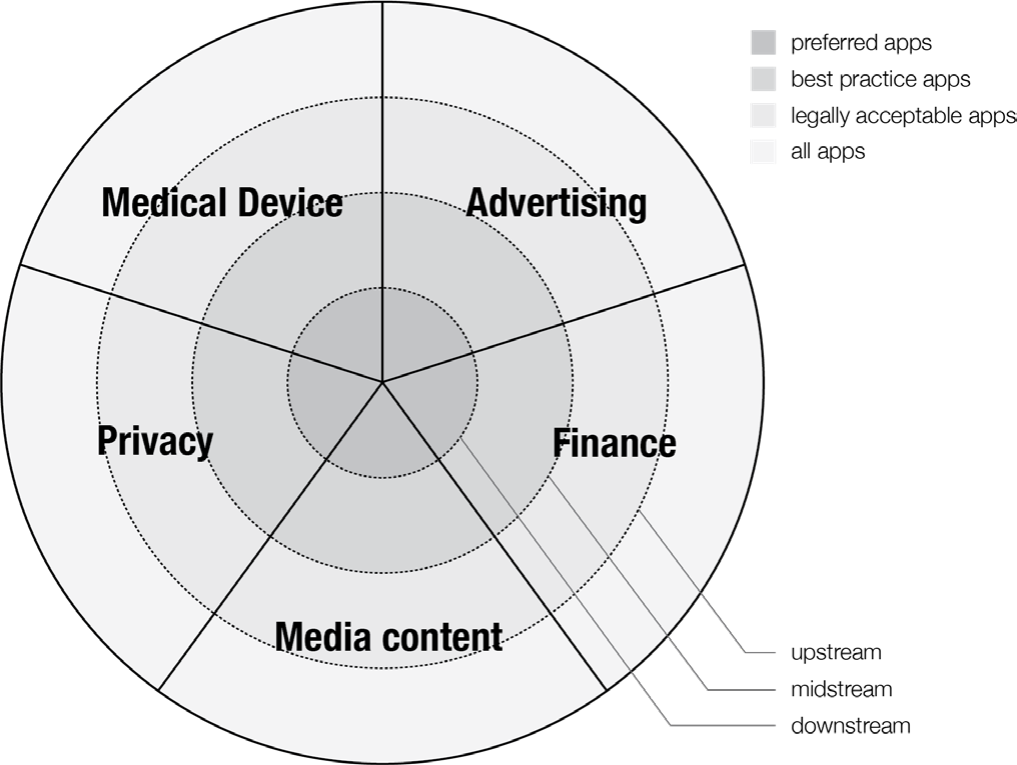
Framework for Regulatory Influence.

Each locus of oversight acts as a semi-permeable barrier, filtering the ‘stream’ of apps and sequentially reducing the size of the app market to which consumers are exposed (Figure 1). The initial population of health apps is filtered through ‘upstream’ government regulation resulting in the availability of legally acceptable apps. ‘Midstream’ policies such as industry self-regulation or app store criteria filter the legally acceptable apps leaving best practice apps. Finally, ‘downstream’ policies (including consumer education, external app certification, and app libraries) evaluate and curate the apps available in the market resulting in a small inner pool of ‘preferred apps.’

Oversight may be siloed and is not necessarily comprehensive. Different aspects of a single app may come under the jurisdiction of multiple upstream regulators, with none of them appearing to take overall responsibility for the product. For example, the US Federal Trade Commission (FTC), which provides upstream protection for consumers’ interests in the marketplace, has produced a document providing “best practice” guidance to help health app developers “build privacy and security into [their] app” [S10a]. The FTC does not provide guidance on health app efficacy and safety, but directs those whose apps are “intended for use in the diagnosis of disease or other conditions, or in the cure, mitigation, treatment or prevention of disease” [S10b] to the FDA “to see if the FDA intends to apply its regulatory oversight for your type of app” [S10b].

Health apps that slip past upstream regulators may also fail to be captured by midstream regulators. For example, commercial app store guidance gives little attention to safety in health apps. The Google Play app store guidelines only alert developers that the store does not “allow … apps that feature medical or health-related functionalities that are misleading or potentially harmful”[S14]^(p9)^ while the Apple iTunes store guidelines simply state that “[m]edical apps that could provide inaccurate data or information, or that could be used for diagnosing or treating patients may be reviewed with greater scrutiny” [S13]^(1.4.1)^.

Downstream regulation is piecemeal and voluntary: developers and consumers may be unaware of it or may choose to ignore it. About half of the midstream and downstream policies had industry origins or partners and may better represent commercial interests.

### What Is the Nature of the Problem of Health App Regulation?

The policies all provided a ‘solution,’ and thus implicitly represented a ‘problem,’ related to mental health app regulation.^36^ We identified a range of problem representations, with some more prominent than others, including: burdensome legislation, barriers to commercial success, and difficulty with consumer choice. Protection of consumers was, in comparison, represented to be a minor problem.

Confusing and burdensome legislation was the dominant problem representation in several government policies (eg, [S5,S8,S11]). These policies sought to “provide [legislative] clarity to industry and [government] staff” [S5]^(p1)^. Other policies represented the problem as the burden of regulatory compliance that fell upon industry, including associated financial costs [S11].

Many upstream policies and both midstream policies were aimed at commercial business interests, representing a problem of fragile commercial reputations:

“Don’t include any hidden or undocumented features in your app…We work hard to make the App Store a trustworthy ecosystem and expect our app developers to follow suit” [S13]^(2.3.1)^.

Other policies (eg, [S15, S10a]) represented legal as well as reputational business problems:

“False or misleading claims, as well as the omission of certain important information, can tick off users and land you in legal hot water” [S9]^(p1)^.

Numerous downstream policies related to tools that consumers might use to identify particular apps, eg, curated app libraries, app certification programs, consumer guidance and education. These policy solutions implied or stated a problem of choice for consumers and health professionals: “with thousands of different mobile applications (apps) out there, trying to choose ones that are reliable and effective can be a daunting task” [S21].

Protecting app users was a less prominent issue within the policy sample, presented only in upstream policies. Three policies [S8,S15,S28] presented loss of privacy as a consumer protection (rather than a business protection) problem. These policies advised app developers to embed “better” [S15] ^(p1)^ privacy practices in their products in order to avoid inadvertently facilitating the loss of “significant amounts of personal [consumer] data” [S7]^(p5)^ that would result in “risks to the … reputation of users of smart devices” [S7]^(p2)^. No policies represented consumer health as problematic although one policy alluded to the possibility, noting “it is not yet clear if and to what extent lifestyle and wellbeing apps could pose a risk to citizens’ health” [S8]. This policy did not propose any related solutions.

### Policy Subjects

**Figure 2:**
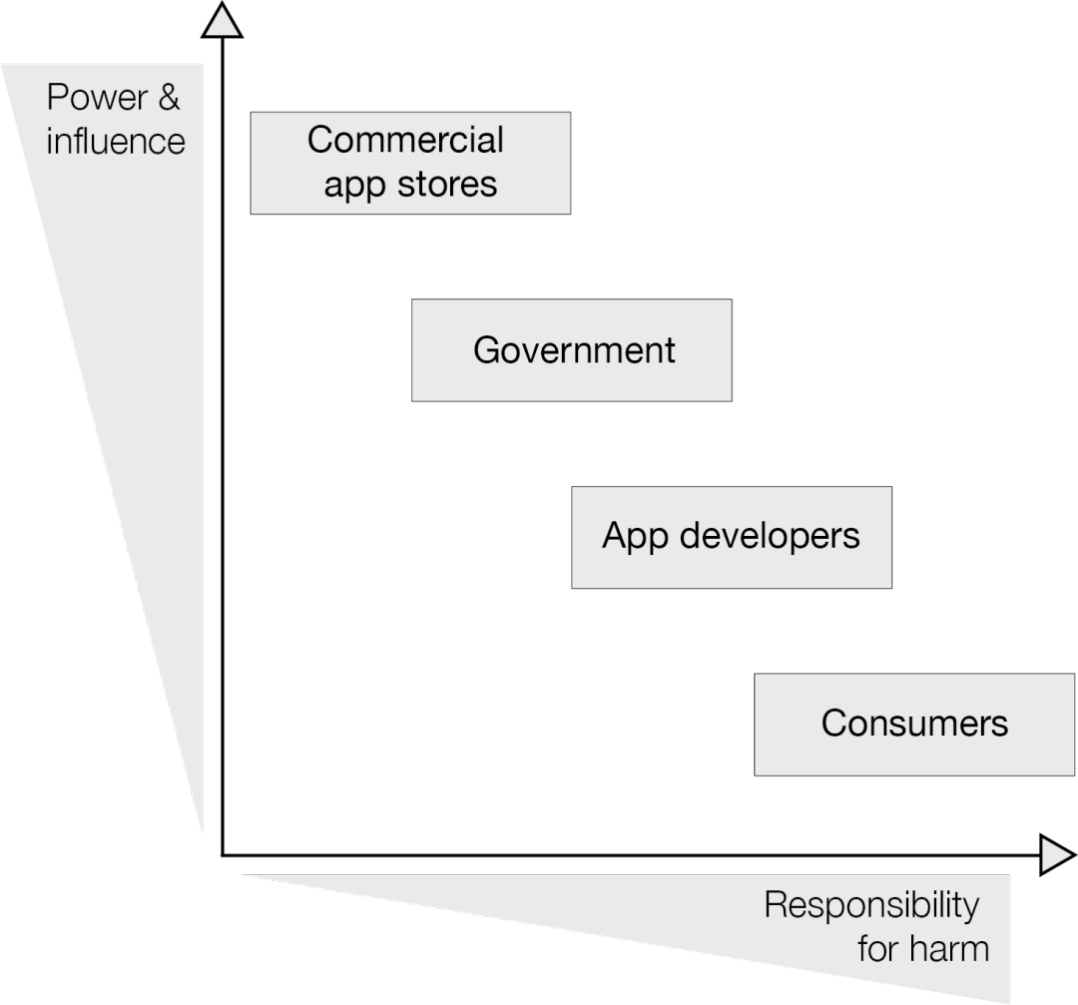
The Relative Power and Influence of Health Policy Target Populations.

Policy authors and subjects referenced in the sampled policies included: app developers, consumers, regulators, app distributors, representative bodies aligned with relevant industries (such as digital advertising and mobile privacy), app library curators (commercial or non-profit entities involved in selecting and presenting a ‘library’ of preferred apps) and additional commercial partners within the so-called ‘mobile ecosystem’ (such as businesses offering tools on digital privacy). Within the policy sample, subjects tended to be ascribed responsibility for outcomes in inverse proportion to their influence over the health apps arena, such that the most commercially and politically powerful subjects (app distributors) were given less responsibility, while less powerful entities (consumers) were given the most responsibility (Figure 2).

**App developers** (also known as app manufacturers [S4] or publishers [S26a]) were often referred to as individuals, typically male. Developers were referenced as “small start-ups” [S7]^(p5)^ or “a large team of experienced programmers”[S13]^(p1)^. They were generally presented positively:

“We. . . honor what you do. We’re really trying our best to create the best platform in the world for you to express your talents” [S4] ^(p2)^.

At the same time, policies frequently referred to inexperienced, “first time app developer[s]”[S4]^(p1)^ who had “little or no prior programming skills” [S7]^(p5)^.

A minor, or “rogue” subjectification of the app developer was “dishonest,” [S13]^(p9)^ frowned upon for attempting to “cheat the system…or manipulate ratings,” [S13]^(p2)^ or “fleecing unsuspecting users” [S12] ^(p16)^ with “expensive apps that try to cheat users with irrationally high prices” [S12]^(p13)^ and for trying to “copy…or unfairly use other people’s work” [S14]^(p4)^. Incompetent developers who produced sub-standard apps were rejected for being likely to annoy “serious developers who don’t want their quality apps to be surrounded by amateur hour” [S13]^(p1)^. Policies promoted the idea that market forces would ensure rogue developers were in the minority: “Their ability to thrive is limited and they are therefore unlikely to cause widespread and systemic consumer harm” [S12]^(p40)^.

Clinical app developers were another sub-category. Policies presented these medical professionals positively as experts driven by ethical principles of beneficence and justice.

**App consumers** discussed within the policies included: patients, carers such as family and friends, and general citizens. Most policies described adults, sometimes specifically parents, occasionally children. Consumers were “confused,”[S18a] “unsuspecting” [S20a]^(p5)^ and “lack[ing] the ability to choose appropriate protection options,”[S11]^(p18)^ yet expected to “be smart about how and where you use your mobile device,”[S23] with the skills and understanding to “know what you are getting into before downloading,”[S50] and the wherewithal to “pay special attention to apps in which you enter personal information”[S20a]. They were often presented as irresponsible, “not tak[ing] the time to read” app privacy policies,[S26a]^(p7)^ Consumers were waiting for developers to tell them what they needed:

“Ensure that you communicate to the customer that a gap exists [in their lives] and that your app can fill it” [S15f]^(6.1.2)^.

A secondary subjectification of app consumers was the “informed, discerning and influential consumer,” who had the power to generate “negative publicity” for the app developer if they lost confidence in the product [S6]^(p2)^.

**Regulators** appeared within policies as ineffective government agencies. For example, regulators described their policies as reactionary to “innovators [who] have begun to develop mobile apps of increasing complexity,” [S4]^(p6)^ positioning themselves as followers rather than leaders. They were unable to easily explain legislation to key stakeholders: “how [regulatory measures] are applied may not always be clear to consumers and industry participants” [S11]^(p15)^.

**App distributers** included Apple’s iTunes app store and Google’s Google Play store. Their subjectification was as “reputable provider[s]” [S23] of “trusted, high-quality…content,”[S14]^(p33)^ providing benefit to consumers through the distribution of health apps.

**App library curators** used a range of tools to evaluate apps. Most used in-house checklists with health professionals conducting reviews. Curators portrayed themselves positively as “dedicated to consumers,” “committed,” and “free of preference or bias” [S22]. They were also useful to industry, creating space within their libraries for “a number of [advertising] opportunities for partners to…get their message out” [S20b].

Policies frequently referred to the **mobile ecosystem**, which included: app owners (if different from app developers); Operating System and mobile device manufacturers; mobile and internet providers; and entities involved in data collection and processing (often referred to as ‘third parties’) such as advertisers, brokers for aggregated data sets, and data analytics companies. The ecosystem was sometimes represented as useful, enabling app developers to see bigger profits via “new aggressive forms of ad delivery” that “create new options for monetization,” and provide consumers with “incredibly informative, relevant and delightful experiences” [S26a] ^p19-20^. Others presented the ecosystem as untrustworthy elements within a connected industry whose “chain of mobile actors is only as strong as its weakest link” [S7]^(p2)^.

###  Responsibility for User Outcomes

Responsibility for user outcomes was allocated to stakeholders at odds with their ability to influence the health app market (Figure 1). Developers were assigned the most responsibility, while simultaneously acknowledged to be just one of multiple relevant elements. For example, “third-party services such as advertising are developing rapidly [and] if integrated by an app developer *without due regard* may disclose significant amounts of personal data” [emphasis added] [S7]^(p5)^. Developers were told they “should be mindful of what ad providers they integrate into their applications: optimizing monetary return should not be the only consideration”[S26a]^(p20)^. Poor user outcomes were the responsibility of ill-informed developers who did“not fully understand[d] the implications” of the data collection and usage practices of their commercial partners. [S26a]^(p7)^.

Consumers were also accorded heavy responsibility for user outcomes. They were expected to take active steps to protect their privacy, for example by choosing apps wisely, [S21] reading privacy policies, [S20a] and securing their mobile device [S23]. However consumers were also expected to “share…progress with friends…or other users of the app community,” [S15a] in order to engender positive health behaviour change.

Governments and industry, including the commercially and politically powerful app stores, were accorded less responsibility for user outcomes; for example consumer protection policies (eg, stamping out overly predatory, coercive or inappropriate practices) were voluntary. Similarly, libraries were assigned no responsibility, despite having the potential to exercise substantial power over developers who wish to be included, and over consumers who use their recommended products.

## Discussion

Our results identified considerable policy activity related to oversight of health app development, distribution and selection for use. Gaps between oversight mechanisms were apparent, at least partly in response to upstream regulators shunting regulatory responsibility among themselves like a ‘hot potato’ that none wished to catch. The dominant regulatory concerns were to clarify and contain the reach of existing legislation, protect commercial reputations, and reduce the consumer burden of app selection. In comparison, there was less regulatory attention to consumer protection. Policies attributed responsibility for outcomes of app use to app developers and app consumers more than to governments and commercial entities including dominant app stores.

Existing upstream regulation is scattered throughout a range of separate sectors; oversight of each regulatory agency is limited, and some apps fall between legislative instruments. Developers may not be aware of all relevant regulations, and hence fail to adhere to their guidance. The most influential gatekeepers for the mental health app market are the midstream operators: multinational technology companies that operate dominant app stores. Other powerful stakeholders within the mobile ecosystem include commercial third parties that buy and process consumer data, whose business strategies inherently conflict with the protection of consumer privacy. Downstream regulation is limited, providing only a flimsy barrier to the flow of apps towards consumer selection, and much of it is commercially-backed, which poses a risk of sponsorship bias.

Consumer interests may be advanced by health apps, but they may also be compromised.^11,12,14,19^ Despite these well-described risks, consumer issues are not the main problem representation within health app policies. Instead, policies describe commercial problems for app developers and others. App developers were positively constructed, and afforded regulatory ‘carrots’ or cautionary tales rather than punitive measures. However, due to their limited influence over the health app market, a regulatory focus on app developers as the policy subjects of regulation is unlikely to afford widespread consumer protection. Instead, we identified large commercial actors in the health app market, namely the app stores, who may be much more effective targets of regulation when it comes to oversight of health apps.

The limited regulatory attention to consumer protection is likely to have a multi-faceted explanation. The nature of health apps means there are multiple types of possible harm to consumers (eg, harms related to user health, privacy, finances, consumer rights). Since each of these is formally overseen by a different government agency, it is difficult to ascribe primary regulatory responsibility to a single body. Even in the United States, which has a legislative mandate for interagency cooperation^41^ there is no central regulator with overall accountability. Agencies may be wary of taking on a regulatory role if their responsibility is not clear, and the huge volume and constantly changing nature of health app products means that regulation of the entire field is simply beyond the capacity of traditional regulatory bodies. Underlying factors contributing to weak government regulation in this area may include strong lobbying from industry and long-term underfunding of government regulatory bodies.^42^

There was a notable lack of policy around health app efficacy, arguably an issue relevant to medical device regulators and consumer protection agencies. Medical device efficacy is important because devices are judged according to whether likely benefits outweigh harms. Medical device agencies are unlikely to play a major role in this issue. For example there has been no FDA enforcement action against health app developers since 2013,^43^ and their proscribed scope now excludes most health apps on the grounds that they are not devices and/or are low risk. A recent review of US regulation and case law related to mental health apps suggests that the potential for user harm will be the primary factor in determining the level of oversight; to date, there was no case law, however, specifically related to harms stemming from mental health apps.^44^ Health app efficacy may also be relevant to consumer protection agencies, because misleading claims of benefit contravene consumer marketing rights. The FTC has recently brought cases against 2 health apps on the grounds of misleading marketing. The cases were settled without a court judgment.^44-46^

## Policy Recommendations

There is a growing literature acknowledging the current limitations of regulatory oversight for health apps, with a range of suggested approaches including stronger regulatory focus from governments^22,42^ and ongoing consumer education for technological literacy.^13^ Strengthening the position of upstream regulators will not only protect consumers but may also foster public confidence in the industry, enabling the market to expand and improve.^22,29,42^ We recommend further efforts to educate consumers about the risks of harm that health apps pose, with suggestions for safe health app use as part of technological literacy programs for app users and clinical prescribers.^13^ However, since consumers have relatively little opportunity to effect change on the overall app market, consumer-focussed solutions should not be the dominant consumer protection strategy. Support for consumer advocacy groups to lobby governments and industry for changes in legislation or industry practices may better support consumer interests. Similarly, instructing consumers to only choose high quality apps may be less useful than adopting better systems for external assessment of health app efficacy and quality.^11^

Regulators should concentrate on entities that have the most influence over the health app market, ie, app stores and other commercial partners within the mobile ecosystem. Currently midstream actors are able to effect significant influence over consumer experiences of health apps (eg, by setting standards for app content and for behaviour of commercial partners who collect and use consumer data.) However, the regulatory trajectory appears to favour well-established, major technology companies in reducing regulatory burden. The FDA has recently introduced a new Pre-Cert pilot program, which will allow selected companies, with a good track-record for quality, to have expedited review of their medical device software, meaning their products can bypass many of the requirements of a full review.^6,23^ Although this field is in need of policy innovation and the Pre-Cert model may help to address the regulatory challenges associated with a large and rapidly iterating market, we suggest that more stringent standards for efficacy, safety, privacy, security, and marketing be imposed on the commercial app distributors and major app developers. We also advocate that the regulator maintain its independence from the industry it aims to regulate; the FDA’s dedicated Digital Health Unit in the medical device center will largely be funded by industry user fees,^6^ creating a conflict of interest, which is not necessarily in the best interests of consumers. Further, industry is implicated in undermining or shaping regulatory authority to its own advantage.^42^ For example, journalists revealed that Apple representatives have been secretly meeting with FDA officials for years, were invited to join the IMDRF working group, but requested their participation be omitted from public records.^47^

One way to improve consumer protection would require a significant shift in regulatory focus: oversight of the developers, app stores, and third-parties involved in the commercialisation of consumer data. The General Data Protection Regulation in Europe represent an advance in transparency and accountability in regards to consumers’ privacy.^48^ However, we recommend systems be put in place to improve transparency over the collection and use of health app data, enable consumer correction of incorrect data, mandate reporting of adverse events, and enforce adherence to acceptable practices as occurred in the consumer credit industry in the United States.^14^

## Limitations

Our case study and purposive sampling approach means that our sample is not comprehensive nor representative of all policies related to health app regulation. This is particularly the case for policies derived from non-English speaking countries and any insights may not be generalizable to these jurisdictions. This is a rapidly moving field, and there may have been new policies released since sampling occurred. Nevertheless, our sampling strategy and policy sample was inclusive of a range of stakeholders and mechanisms across sectors and countries.

Although our analysis was carried out with particular attention to the Australian mental health app market, our policy sample drew heavily from source material from other jurisdictions and entities with a global influence and few were specific to mental health. As a result, our findings are likely transferable to the regulation of health app markets internationally, particularly for other developed countries with similar regulatory landscapes.

## Conclusion

Enthusiasm for health apps appears to be running ahead of oversight designed to protect the consumers who are encouraged to use them. This appears to be based on assumptions that they will deliver substantially more health and economic benefits than harms. These assumptions may be influenced by commercial bias; hence, we urge regulators and policy-makers to be mindful of the influence over consumer outcomes wielded by commercial entities in the health app market. We encourage consumer advocacy groups, app developers, health professionals and governments to work together, avoiding the ‘hot potato’ phenomenon which may create regulatory gaps, and prioritising a stronger regulatory focus on powerful commercial entities to improve consumer privacy and safety.

## Acknowledgments

The authors would like to thank Lorris Williams, MFA for figure design.

## Ethical issues

This study did not require ethics committee approval because it did not involve research on human, human data, or human tissue.

## Competing interests

Authors declare that they have no competing interests.

## Authors’ contributions

LP participated in the study design, conducted data collection and analysis and drafted the manuscript. QG conceived of the study, participated in its design, data collection and analysis and conducted major revisions. LB, DG, and MR participated in study design and analysis and revised the manuscript. All authors read and approved the final manuscript.

## Authors’ affiliations

^1^Charles Perkins Centre, Faculty of Medicine and Health School of Pharmacy, The University of Sydney, NSW, Sydney, Australia. ^2^NDIS Quality and Safeguards Commission, Penrith, NSW, Australia. ^3^Critical and Ethical Mental Health Research Group, Robinson Research Institute, The University of Adelaide, Adelaide, SA, Australia. ^4^Discipline of Public Health, Flinders University, Adelaide, SA, Australia. ^5^Faculty of Nursing, University of Toronto, Toronto, ON, Canada.

## Funding

This work was supported by the Australian Communications Consumer Action Network (ACCAN). The operation of the Australian Communications Consumer Action Network is made possible by funding provided by the Commonwealth of Australia under section 593 of the *Telecommunications Act 1997*. This funding is recovered from charges on telecommunications carriers.

## Supplementary files

Supplementary file 1 contains Sampled policies.

## Key messages

**Implications for policy makers**

- There are gaps in the regulatory framework for health apps, at least partly because the different sectors involved (medical device, advertising, finance, media content, privacy) are relatively siloed, with no single sector holding comprehensive oversight.
- There is a lack of regulatory focus on consumer protection for app users, with regulation instead concentrating on reducing burdens of consumer choice.
- Regulatory policies tend to ignore regulatory responsibilities of commercial app stores, which serve as distributers, even though the app stores arguably wield more power in the health app arena than policymakers and individual developers.
- Policy-makers in different sectors should work together to develop an overarching regulatory framework for health apps, with a focus on consumer protection.
- Governments should put pressure on commercial app stores to change practices in favour of protecting consumer privacy and safety.

**Implications for public**

Consumers should be aware that the current regulatory framework is inadequate, meaning that some publicly available health apps may harm their health, finances or privacy. Consumers and advocacy groups should lobby governments and industry for changes in legislation and industry practices that better supports consumer privacy and health safety.
