# Supplementary material for: The "Hot Potato" of Mental Health App Regulation: A Critical Case Study of the Australian Policy Arena
Source: Int J Health Policy Manag. 2018 Dec 16;8(3):168–76. doi: 10.15171/ijhpm.2018.117 (PMC6462196; doi:10.15171/ijhpm.2018.117)
Supplement: Supplementary file 1 — contains Sampled policies. [file ijhpm-8-168-s001.pdf]

Supplementary File 1

Sampled Policies

Code used in text	***Title* (Author, Year)**	**Author Type**	**Location**	**Sector**
	UPSTREAM			
S1	*Regulation of Medical Software and Mobile Medical 'Apps'* [1]	Government	Australia	Health
S2	*Software as a Medical Device (SaMD)*[2]	Government	International	Health
S3	*Guidance: Medical Device Stand-alone Software Including Apps (including IVDMDs)*[3]	Government	UK	Health
S4	*Mobile Medical Applications: Guidance for Industry and Food and Drug Administration Staff* [4]	Government	USA	Health
S5	*General Wellness: Policy for Low Risk Devices*[5]	Government	USA	Health
S6	*Mobile Privacy: A Better Practice Guide for Mobile App Developers*[6]	Government	Australia	Privacy
S7	*Opinion 02/2013 on Apps on Smart Devices*[7]	Government	Europe	Privacy

S8	*Commission Staff Working Document on the Existing EU Legal Framework Applicable to Lifestyle and Wellbeing Apps*[8]	Government	Europe	Privacy, Commerce, Advertising
S9	*Marketing Your Mobile App: Get it Right from the Start*[9]	Government	USA	Trade
S10a, S10b	*Mobile Health App Developers: FTC Best Practices*[10][S10a] and accompanying *Mobile Health Apps Interactive Tool*[11][S10b]	Government	USA	Trade
S11	*Mobile Apps: Emerging Issues in Media and Communications (Occasional paper 1)*[12]	Government	Australia	Media
S12	*App Purchases by Australian Consumers on Mobile and Handheld Devices: Inquiry Report*[13]	Government	Australia	Commerce
MIDSTREAM				
S13	*App Store Review Guidelines*[14]	Industry	International	Technology
S14	*Developer Policy Center*[15]	Industry	USA	Technology
DOWNSTREAM				
S15a - S15g	*VicHealth's Top 10 Tips for Choosing a Healthy Living App; Guidelines for Creating Healthy Living Apps*[16] [S15a]and related references:[17][S15b],[18][S15c],[19][S15d],[20][S15e],[21][S15f],[22][S15g]	Government	Australia	Health
S16	*Mobile App Rating Scale (MARS)*[23]	University	Australia	Health

S17a, S17b	*How to Choose a Health App*[24][S17a] and related reference[25][S17b]	Health System	New Zealand	Health
S18a - S18c	*myhealthapps.net*[26][S18a] and related references[27][S18b],[28][S18c]	Industry	UK	Health
S19	*Safety and Quality Strategy in Mobile Health Apps*[29]	Government	Spain	Health
S20a, S20b	*Doctor's Guide to Choosing Health Apps that Really Work*[30][S20a] and related reference[31][S20b]	Industry	USA	Health
S21	*Choose Wisely: Selecting Mobile Health Apps*[32]	Health System	USA	Health
S22	*PsyberGuide*[33]	Not-for-profit	USA	Health
S23	*Stay Smart Online: Mobile Devices*[34]	Government	Australia	Privacy
S24	*TRUSTed Apps Privacy Certification*[35]	Industry	USA	Privacy
S25	*Mobile and Privacy: Privacy Design Guidelines for Mobile Application Development*[36]	Industry	International	Tele-communications
S26a, S26b	*Mobile App Advertising Guidelines: A Framework for Encouraging Innovation While Protecting User Privacy*[37][S26a] and related reference[38][S26b]	Industry	USA	Advertising
S27	*A Guide to Apps & In-app Purchases*[39]	Government	Australia	Media

S28	*Applications of Self-Regulatory Principles to the Mobile Environment*[40]	Industry	USA	Advertising
S29a - S29d	*Best Practice Guideline – Responsible Marketing Communications in the Digital Space*[41][S29a] and related references [42][S29b],[43][S29c], [44][S29d]	Industry	Australia	Advertising

References

1. Therapeutic Goods Administration (TGA). Regulation of medical software and mobile medical 'apps' Canberra, ACT: Australian Government Department of Health; 2013 [cited 2015 October 22]. Available from: https://www.tga.gov.au/node/4316.
2. International Medical Device Regulators Forum. Software as a Medical Device (SaMD): Key definitions (2013); Possible framework for risk categorization and corresponding considerations (2014); application of quality management systems (2015): IMDRF; 2013 2014 2015 [cited 2017 March 1]. Available from: http://www.imdrf.org/workitems/wi-samd.asp.
3. Medicines and Healthcare products Regulatory Agency (MHRA). Medical device stand-alone software including apps (including IVDMDs). London: MHRA, 2016.
4. U.S. Food and Drug Administration (FDA). Mobile medical applications: Guidance for industry and Food and Drug Administration staff. Silver Spring, MD: FDA, 2015.
5. U.S. Food and Drug Administration (FDA). General wellness: Policy for low risk devices. Rockville, MD: U.S. Food and Drug Administration, 2016.
6. Office of the Australian Information Commissioner (OAIC). Mobile privacy: A better practice guide for mobile app developers. Canberra, ACT: Australian Government, 2014.
7. Article 29 Data Protection Working Party. Opinion 02/2013 on apps on smart devices. Brussels, Belgium: European Commission, 2013.
8. European Commission. Commission Staff Working Document on the existing EU legal framework applicable to lifestyle and wellbeing apps. Brussels, Belgium: 2014.
9. Federal Trade Commission (FTC). Marketing your mobile app: Get it right from the start. Washington, DC: 2013.

10. Federal Trade Commission (FTC). Mobile health app developers: FTC best practices Washington, DC: Federal Trade Commission, 2016 November 1.

11. Federal Trade Commission (FTC). Mobile health apps interactive tool. Washington, DC: 2016 November 1.

12. Australian Communications and Media Authority (ACMA). Mobile apps: Emerging issues in media and communications. Canberra, ACT: 2013.

13. Commonwealth Consumer Affairs Advisory Council (CCAAC). App purchases by Australian consumers on mobile and handheld devices: Inquiry report. Canberra, ACT: CCAAC, 2013.

14. Apple Inc. App Review Cupertino, CA: Apple Inc.; 2015 [cited 2017 February 24]. Available from: https://developer.apple.com/support/app-review/.

15. Google Play. Developer Policy Center Mountain View, CA: Google Inc.; 2016 [cited 2016 October 27]. Available from: https://play.google.com/about/developer-content-policy/.

16. VicHealth. VicHealth's top 10 tips for choosing a healthy living app. Carlton, Victoria: VicHealth, 2015 September 29.

17. VicHealth: caution needed when downloading health apps [press release]. Carlton, Victoria: VicHealth, September 14 2015.

18. VicHealth. Health Living Apps Guide Carlton, Victoria: VicHealth; 2016 [updated October 26; cited 2016 October 31]. Available from: https://www.vichealth.vic.gov.au/media-and-resources/vichealth-apps/healthy-living-apps.

19. VicHealth ratings guide shows which health and wellbeing apps work best [press release]. Carlton, Victoria: VicHealth, October 26 2016.

20. VicHealth. Selecting, reviewing and rating healthy living apps - our process. Carlton, Victoria: VicHealth, October 25 2016.

21. dialogue consulting. Guidelines for creating health living apps. Melbourne, Australia: Deakin University, 2015.

22. VicHealth. Is there an (effective) app for that? Carlton, Victoria: VicHealth, 2016 January 14. Report No.

23. Stoyanov SR, Hides L, Kavanagh DJ, Zelenko O, Tjondronegoro D, Mani M. Mobile app rating scale: a new tool for assessing the quality of health mobile apps. JMIR mHealth uHealth. 2015;3(1):e27.

24. Health Navigator NZ. How to choose a health app Auckland, New Zealand: Health Navigator; 2016 [updated August 31; cited 2016 October 11]. Available from: http://www.healthnavigator.org.nz/app-library/h/health-apps-how-to-choose/.

25. Health Navigator NZ. App library Auckland, New Zealand: Health Navigator; 2016 [updated October 10; cited 2016 October 11]. Available from: http://www.healthnavigator.org.nz/app-library/.

26. myhealthapps.net. my health apps: tried and tested by people like you London: Patient View Ltd; [cited 2016 October 11]. Available from: http://myhealthapps.net/.

27. myhealthapps.net. The myhealthapps directory 2015-2016. London: 2015.

28. my health apps. Health apps: Towards a balanced life. London: my health apps, 2014.

29. Agencia de Calidad Sanitaria de Andalucía. Safety and quality strategy in mobile health apps: Agencia de Calidad Sanitaria de Andalucía; 2012 [cited 2017 April 21]. Available from: http://www.calidadappsalud.com/en/.

30. Misra S. Doctors' guide to choosing health apps that really work. everydayhealthcom. 2015 July 29.

31. iMedicalApps. iMedicalApps + Medpage Today U.S.A.: Everyday Health Property; 2017 [cited 2017 March 1]. Available from: iMedicalApps.com.

32. Navy and Marine Corps Public Health Center (NMCPHC). Choose wisely: selecting mobile health apps. Portsmouth, VA: NMCPHC, 2014 November 28. Report No.

33. PsyberGuide. PsyberGuide Rutherford, CA: One Mind Institute; [cited 2016 October 11]. Available from: http://psyberguide.org/.

34. Attorney-General's Department. Stay smart online: Mobile devices Canberra, ACT: Australian Government; 2017 [cited 2017 February 24]. Available from: https://www.staysmartonline.gov.au/mobile-devices.

35. TRUSTe. TRUSTed Apps Privacy Certification San Francisco, CA: TRUSTe Inc.; 2016 [cited 2016 September 13]. Available from: https://www.truste.com/business-products/trusted-apps/.

36. GSMA. Mobile and privacy: privacy design guidelines for mobile application development. London: GSMA, 2012.

37. Lookout. Mobile app advertising guidelines. San Francsico, CA: 2012.

38. Lookout. Lookout App Security San Francsico, CA: Lookout Inc.; [cited 2016 November 2]. Available from: https://www.lookout.com/products/app-security.

39. Australian Communications and Media Authority (ACMA). A guide to apps & in-app purchases Canberra, ACT: Australian Government; 2016 [cited 2017 February 24]. Available from: http://www.acma.gov.au/Citizen/Phones/Mobile/Content-and-services/apps-and-in-apps-purchases-a-guide-for-consumers.

40. Digital Advertising Alliance (DAA). Application of self-regulatory principles to the mobile environment. USA: 2013.

41. Australian Association of National Advertisers (AANA). Best practice guideline - responsible marketing communications in the digital space. Sydney, NSW: 2013.

42. Australian Association of National Advertisers (AANA). Code of Ethics. Sydney, NSW: 2016 January.

43. Australian Association of National Advertisers (AANA). Code of Ethics - practice note. Sydney, NSW: 2016 January.

44. Australian Association of National Advertisers (AANA). Native Advertising Principles Sydney, NSW: 2015 November.
